# The therapeutic effect of N‐acetylcysteine as an add‐on to methadone maintenance therapy medication in outpatients with substance use disorders: A randomized, double‐blind, placebo‐controlled clinical trial

**DOI:** 10.1002/brb3.2823

**Published:** 2022-11-30

**Authors:** Fateme Padoei, Peyman Mamsharifi, Pooya Hazegh, Homa Boroumand, Fatemeh Ostadmohammady, Samira Abbaszadeh‐Mashkani, Hamid Reza Banafshe, Amir Hassan Matini, Amir Ghaderi, Somayeh Ghadami Dehkohneh

**Affiliations:** ^1^ Student Research Committee Kashan University of Medical Sciences Kashan Iran; ^2^ Department of Psychology Allameh Tabataba'i University Tehran Iran; ^3^ Department of Psychiatry Kashan University of Medical Sciences Kashan Iran; ^4^ Drug abuse treatment center Kashan University of Medical Sciences Kashan Iran; ^5^ Physiology Research Center Kashan University of Medical Sciences Kashan Iran; ^6^ Department of Clinical Pathology Kashan University of Medical Sciences Kashan Iran; ^7^ Department of Addiction studies, School of Medical Kashan University of Medical Sciences Kashan Iran; ^8^ Clinical Research Development Unit‐Matini/Kargarnejad Hospital Kashan University of Medical Sciences Kashan Iran; ^9^ Department of Pharmacy, Acharya BM ready college of Pharmacy Rajiv Gandhi University of Health Sciences Bangalore Karnataka India

**Keywords:** metabolic biomarker, methadone maintenance treatment, N‐acetylcysteine, psychological status, substance use disorder

## Abstract

**Objective:**

Patients with substance use disorders (SUD) under methadone maintenance therapy (MMT) are susceptible to a number of complications (psychological and metabolic disorders). Evidence studies have shown the roles of the glutamatergic system in addiction. N‐Acetylcysteine (NAC) enhances extracellular glutamate, and is effective in the treatment of neuropsychiatric disorders. We assessed oral NAC as an add‐on to MMT medication for the treatment of SUD.

**Methods:**

In the current randomized, double‐blind, placebo‐controlled clinical trial, outpatients with SUD under MMT who were 18–60 years old received 2400 mg/day NAC (*n* = 30) or placebo (*n* = 30) for 12 weeks. Psychological status and metabolic biomarkers were assessed at baseline and the end of the trial.

**Results:**

Compared with the placebo group, NAC treatment resulted in a significant improvement in depression score (β −2.36; 95% CI, −3.97, −0.76; *p* = .005), and anxiety score (β −1.82; 95% CI, −3.19, −0.44; *p* = .01). Furthermore, NAC treatment resulted in a significant elevation in total antioxidant capacity levels (β 72.28 mmol/L; 95% CI, 11.36, 133.19; *p* = .02), total glutathione (GSH) levels (β 81.84 μmol/L; 95% CI, 15.40, 148.28; *p* = .01), and a significant reduction in high‐sensitivity C‐reactive protein levels (β −0.89 mg/L; 95% CI, −1.50, −0.28; *p* = .005), and homeostasis model of assessment‐insulin resistance (β −0.33; 95% CI, −0.65, −0.009; *p* = .04), compared with the placebo group.

**Conclusion:**

In the current study, improvement in depression and anxiety symptoms as well as some metabolic profiles with NAC treatment for 12 weeks in outpatients with SUD under MMT was detected.

## Graphical abstract

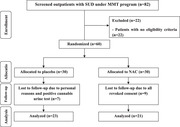

## 1 INTRODUCTION

Opioid use disorder (OUD) is a growing issue globally (Jayawardana et al., 2021). In Iran, the prevalence of this condition is increasing and is almost three times greater than the global prevalence (Amin‐Esmaeili et al., 2016). Methadone has been identified as an effective maintenance drug to treat OUD (Khazaee‐Pool et al., 2018). Methadone maintenance treatment (MMT) enhances mental and physical well‐being, life quality, and psychosocial functioning in patients with OUD (Dolan et al., 2003). Despite carrying out practical implementations of MMT, numerous challenges and questions remained obscure. However, some evidence‐based studies showed OUD correlated with the up‐regulation of inflammatory cytokines and oxidative stress mediators, immune system dysfunction, lipid peroxidation, increased production of reactive species, and DNA damage (Salarian et al., 2018; Vallecillo et al., 2018). Moreover, Sadava et al. (1997) reported that MMT is associated with metabolic disorders similar to type 2 diabetes mellitus and obesity in animal models. Methadone therapy also might link with psychological disturbances such as depression, anxiety, and sleep problems (Callaly et al., 2001).

Pharmacologic treatment options for several SUD (e.g., opioids, stimulants, cannabis, alcohol, phencyclidine and other hallucinogens, inhalants, sedatives, anxiolytics, or hypnotics; tobacco and other or unknown substances) are limited. NAC is a sulfur‐containing agent that is widely studied for its anti‐inflammatory and anti‐oxidant impact (Ghaderi et al., 2019). Because of the potent scavenging activities against various free radicals, NAC is a medicine that is widely used to treat acetaminophen toxicity and as a mucolytic compound (Hans et al., 2022; Tenório et al., 2021). NAC can have anti‐inflammatory and anti‐oxidative impact by enhancing concentration of endogenous glutathione and reducing mRNA expression of pro‐inflammatory mediators (Tenório et al., 2021). A recent meta‐analysis documented that the prescription of NAC could have a beneficial influence on inflammation and body's anti‐oxidative defense system in humans (Faghfouri et al., 2020). Because MMT is associated with raised inflammation and oxidative stress, NAC supplementation can be used to treat metabolic dysfunction caused by MMT program (Ward et al., 2020). Additionally, the results of human and animal studies showed that NAC supplementation could improve metabolic impairments, including dyslipidemia and hyperglycemia (Rani et al., 2020; Villagarcía et al., 2018).

NAC treatment appears tolerable, safe, and affordable (Deepmala et al., 2015). Regarding the antioxidant effect of NAC, it is supposed that NAC supplementation might be favorable in patients under MMT. Accordingly, this study was performed to evaluate the effect of NAC consumption on metabolic profiles and psychological status in outpatients with SUD under MMT program.

## 2 METHODS

### 2.1 Trial design and setting

This was a 12‐week, randomized, double‐blind, placebo‐controlled, parallel‐group clinical trial of NAC in participants with SUD under MMT who were referred to a university‐affiliated outpatient SUD clinic (Soltan Mirahmad) in Kashan, Iran. The Soltan Mirahmad clinic is a center that is certified for the treatment and diagnosis of SUD. The personnel include a Ph.D. addiction expert, a general physician, an experienced psychiatrist, well‐trained nurses, and a clinical psychologist. The World Medical Association Code of Ethics (Declaration of Helsinki) was considered by the Research Ethics Committee of Kashan University of Medical Sciences for study approval **(IR.KAUMS.MEDNT.REC.1399.017)**, and the study was registered at the Iranian Registry of Clinical Trials (www.irct.ir; IR**CT20170420033551N10)**. Before obtaining written informed consent, it was explained to the patients that withdrawing from the clinical trial would not affect their relationship with their healthcare provider.

### 2.2 Inclusion/exclusion criteria

Inclusion criteria: (1) SUD as diagnosed by DSM‐IV‐TR scales, (2) patients who received MMT, and (3) patients who were between 18 and 60 years.

Exclusion criteria: (1) taking of anti‐oxidant and anti‐inflammatory supplements during the last 3 months, (2) hypertension, (3) hypothyroidism and hyperthyroidism, (4) positive urine test for methamphetamine (MA) and cannabis, and (5) unwillingness to cooperate.

### 2.3 Study design and participants

In this trial, participants were assigned to receive either NAC (*n* = 30) or placebo (*n* = 30) for 12 weeks in patients with SUD under MMT. Participants were randomized to receive either 12 weeks of oral NAC (2400 mg/day) or matched placebo, delivered as a take‐home medication. Due to lack of information about the appropriate dosage of NAC for patients with SUD under MMT, we used the treatment duration and dose of NAC based on a previous study in individuals with cannabis use and tobacco use disorder (Gray et al., 2017; Prado et al., 2015). Therefore, the selected dosage of NAC treatment has been demonstrated to be well‐tolerated. It is, however, conceivable that treatment for a longer period would yield better results. After dividing participants were equally randomized, the case group received 600 mg, four times a day, (2400 mg/day) NAC (Avicenna Pharmaceutical Company, Tehran, Iran) and the placebo group received a placebo. All patients received methadone in the form of syrup. There were no changes in dosages and type of prescribed therapy during the study period.

### 2.4 Safety reporting

Adverse events are defined as any undesirable experience occurring to patients during the trial, whether or not related to NAC. The patients were entirely justified about the side effects of NAC or for taking medication and placebo. All patients were requested to inform investigators about any complaints and adverse events during the clinical trial. The signs were recorded at the beginning and each session. Also, possible adverse effects were recorded via telephone call every week and the expert physician was responsible for discontinuing or continuing the drugs.

### 2.5 Randomization and blinding

Randomization was done using computer‐generated random numbers (Stat Trek software) by trained staff at the Soltan Mirahmad Clinic (Kashan, Iran). Allocation concealment was done using sequentially numbered, sealed, opaque packages. Both randomization and allocation were carried out by independent persons who were not involved elsewhere in the clinical trial. Physicians, patients, nurses responsible for referring the patients, the statistician, also the investigators who rated the patients and administered the drugs, were all blinded to the allocation. Placebo tablets were identical to NAC tablets in color, shape, size, and odor. They were all kept in identical containers and were administered by an investigational drug pharmacist.

### 2.6 Assessment of outcome

Psychological status is an important indicator of the health status in patients with SUD under MMT. So, psychological status was considered as the primary outcome and metabolic biomarkers as the secondary outcome.

### 2.7 Psychological measures

Beck's Depression Inventories (BDI) was used to assess the levels of depression. BDI was determined using a self‐compiled questionnaire of 21 items in multiple‐choice format (Beck et al., 1961). Anxiety was measured by Beck Anxiety Inventory (BAI‐21) questionnaire developed by Beck et al. (1988) in order to assess the anxiety level. Sleep quality was defined by using Pittsburgh Sleep Quality Index (PSQI). It differentiates poor from good sleep quality by measuring seven components of sleep over the last month (Buysse et al., 1989).

### 2.8 Metabolic measures

At baseline and the end of the trial, 10 ml fasting blood was collected from each patient at the Kashan laboratory (Iran) in the early morning after an overnight fast. Blood was collected in two separate tubes: (1) one without EDTA to separate the serum to determine serum insulin, fasting plasma glucose (FPG), lipid profiles, and high sensitivity C‐reactive protein (hs‐CRP) concentration and (2) another one containing EDTA to examine plasma nitric oxide (NO), total antioxidant capacity (TAC), total glutathione (GSH), and malondialdehyde (MDA). Blood samples were immediately centrifuged (Hettich D‐78532, Tuttlingen, Germany) at 3500 rpm for 10 min to separate plasma and serum. Then, the samples were stored at −80°C before analysis at the Kashan laboratory. Serum insulin status was measured using an ELISA kit (Diametra, Italy) with inter‐ and intra‐assay coefficient variances (CVs) of 4.5% and 2.6%, respectively. Quantitative insulin sensitivity checks index (QUICKI) and homeostasis model of assessment‐insulin resistance (HOMA‐IR) were calculated using the established formulas (Pisprasert et al., 2013). Enzymatic kits (Pars Azmun, Iran) were used to measure FPG and lipid profiles with intra and inter‐assay CVs less than 5%. hs‐CRP status was measured using an ELISA kit (LDN, Germany) with intra and inter‐assay CVs less than 7%. The NO status was measured using the Griess method (Tatsch et al., 2011). TAC status was assessed using the ferric reducing antioxidant power method (Benzie & Strain, 1996), GSH using Beutler et al. method (Beutler & Gelbart, 1985), and MDA concentrations by the thiobarbituric acid reactive substances spectrophotometric test with intra and inter‐assay CVs less than 5% (Janero, 1990).

### 2.9 Statistical analysis

*p*‐Values < .05 were considered statistically significant. The Kolmogorov–Smirnov test was done to assess the normality of data. Statistical analyses were done using the Statistical Package for Social Science version 18 (SPSS Inc., Chicago, IL, USA). Descriptive statistics were calculated for demographic variables. To detect the differences in anthropometric scales among two intervention groups, we used the independent‐samples Chi‐square and *t*‐test analyses. Multiple linear regression models were used to determine treatment effects on trial outcomes (psychological status and metabolic biomarkers) after adjusting for confounding variables. The effect sizes were presented as the mean differences with 95% confidence intervals.

### 2.10 Sample size

We did not detect a similar trial about the effects of NAC on psychological status and metabolic biomarkers in patients with SUD under MMT for determining the sample size based on the primary outcome. Hence, the sample size was calculated based on the effects of oral NAC treatment on the status of inflammatory markers in continuous ambulatory peritoneal dialysis patients. Type one (*α*) and type two errors (*β*) were defined as .05 and .20 (power = 80%), respectively. Based on a previous study (Purwanto & Prasetyo, 2012), we used a standard deviation (SD) of 1.32 and 1.17 for NAC and placebo groups, and a mean difference (d) of 1.05, considering CRP as the key scales. We needed 23 participants in each group. Assuming a dropout of 7 subjects per group, the sample size was considered to be 30 participants in each group.

## 3 RESULTS

**FIGURE 1:**
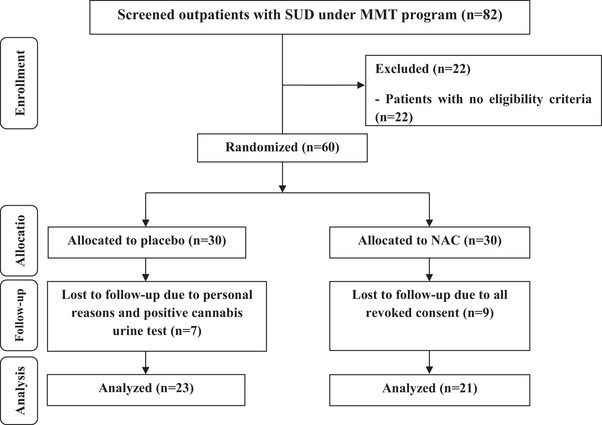
Summary of patient flow diagram

Out of 82 screened outpatients with SUD under MMT program, 60 individuals were enrolled in the clinical trial and randomly assigned to both treatment and control groups to receive NAC or placebo, respectively (30 individuals to each group). In the control group, seven people discontinued the clinical trial due to personal reasons, and positive cannabis urine tests. In the intervention group, nine people revoked their consent. Hence, 44 individuals (intervention [*n* = 21], and control [*n* = 23]) were analyzed. The CONSORT flow diagram on the enrollment of individuals in the present study is demonstrated in Figure 1. No serious adverse effects were reported in this clinical trial following the consumption of NAC supplements in outpatients with SUD under MMT program. Two individuals in the NAC group reported abdominal pain but this side effect did not result in the discontinuation of the clinical trial. No significant difference was observed in the participants in the two treatment arms regarding baseline demographic characteristics of the study (Table 1).

**TABLE 1:** Patient characteristics with SUD under MMT

	Placebo (*n* = 23)^a^	NAC (*n* = 21)^a^	*p* ^b^
Age (y)	47.3 ± 8.1	45.8 ± 9.0	.55
Age first drug use (y)	18.6 ± 3.9	19.5 ± 4.6	.52
Education (%)			
Illiterate	2 (8.7)	1 (4.8)	
Elementary	7 (30.4)	5 (23.8)	
Intermediate	6 (26.1)	8 (38.1)	.82^c^
Diploma	7 (30.4)	5 (23.8)	
High educated	1 (4.3)	2 (9.5)	
Marital status (%)			
Single	8 (34.8)	5 (23.8)	
Married	10 (43.5)	12 (57.1)	.63^c^
Widow/divorced	5 (21.7)	4 (19.0)	
Job (%)			
Unemployed	6 (26.1)	6 (28.6)	
Employed	1 (4.3)	2 (9.5)	.75^c^
Others	16 (69.6)	13 (61.9)	
Duration of MMT (y)	10.6 ± 4.4	8.9 ± 3.8	.18
MMT dose (ml/day)	23.2 ± 10.0	24.7 ± 11.6	.65

^a^ Data are mean ± SDs and percentage.

^b^ Obtained from independent *t*‐test.

^c^ Obtained from Pearson Chi‐square test.

### 3.1 Psychological status

Compared with the control group, NAC significantly reduced the score of depression level (β −2.36; 95% CI, −3.97, −0.76; *p* = .005) and the score of anxiety level (β −1.82; 95% CI, −3.19, −0.44; *p* = .01), respectively. In addition, no significant differences were shown among the two groups in PSQI score (Table 2).

**TABLE 2:** Means (± SD) of psychological status at baseline and after the 12‐week intervention in outpatients with SUD under MMT

Variables	Placebo (*n* = 23)		NAC (*n* = 21)		Difference in outcome measures between NAC and placebo treatment groups^a^	
	Baseline	Week 12	Baseline	Week 12	β (95% CI)	*p* ^b^
BDI	22.8 ± 7.4	24.9 ± 7.4	24.8 ± 4.9	22.5 ± 5.0	−2.36 (−3.97, −0.76)	.005
BAI	14.8 ± 5.3	15.7 ± 4.4	16.7 ± 5.2	15.3 ± 5.1	−1.82 (−3.19, −0.44)	.01
PSQI	3.4 ± 1.3	3.7 ± 1.3	3.7 ± 1.3	3.2 ± 1.2	−0.60 (−1.25, 0.05)	.07

*Note*: Data are mean ± SDs.

Abbreviations: BDI, Beck Depression Inventory; BAI, Beck Anxiety Inventory; PSQI, Pittsburgh Sleep Quality Index.

^a^ “Outcome measures” refers to the change in values of measures of interest between baseline and week 12. β (difference in the mean outcomes measures between treatment groups [NAC group = 1 and placebo group = 0]).

^b^ Obtained from multiple regression models.

### 3.2 Metabolic profiles

NAC significantly decreased HOMA‐IR (β −0.33; 95% CI, −0.65, −0.009; *p* = .04), compared with the placebo group. In addition, NAC resulted in a significant reduction in hs‐CRP (β −0.89 mg/L; 95% CI, −1.50, −0.28; *p* = .005), and a significant increase in GSH levels (β 81.84 μmol/L; 95% CI, 15.40, 148.28; *p* = .01), and TAC levels (β 72.28 mmol/L; 95% CI, 11.36, 133.19; *p* = .02). There was no significant effect of NAC on FPG, insulin, QUICKI, total cholesterol, triglycerides, very‐low‐density lipoprotein (VLDL), LDL, and high‐density lipoprotein (HDL) cholesterol, NO, and MDA levels (Table 3).

**TABLE 3:** Means (± SD) of metabolic biomarkers at baseline and after the 12‐week intervention in outpatients with SUD under MMT

Variables	Placebo (*n* = 23)		NAC (*n* = 21)		Difference in outcome measures between NAC and placebo treatment groups^a^	
	Baseline	Week 12	Baseline	Week 12	β (95% CI)	*p* ^b^
FPG (mg/dl)	92.4 ± 15.7	91.2 ± 20.1	91.8 ± 12.2	84.5 ± 11.1	−5.56 (−12.88, 1.74)	.13
Insulin (μIU/ml)	11.9 ± 1.9	11.9 ± 1.7	12.3 ± 1.9	11.3 ± 1.2	−0.45 (−1.45, 0.54)	.36
HOMA‐IR	2.7 ± 0.6	2.6 ± 0.5	2.8 ± 0.6	2.3 ± 0.4	−0.33 (−0.65, −0.009)	.04
QUICKI	0.32 ± 0.01	0.33 ± 0.01	0.32 ± 0.01	0.33 ± 0.006	0.006 (0.0001, 0.01)	.05
Triglycerides (mg/dl)	166.7 ± 23.1	167.3 ± 35.9	156.8 ± 55.0	151.0 ± 49.3	−13.24 (−40.19, 13.71)	.32
VLDL‐cholesterol (mg/dl)	33.3 ± 4.6	33.4 ± 7.1	31.3 ± 11.0	30.2 ± 9.8	−2.64 (−8.04, 2.74)	.32
Total cholesterol (mg/dl)	183.3 ± 25.1	177.1 ± 41.4	167.9 ± 47.3	150.3 ± 40.6	−17.2 (−41.09, 6.68)	.15
LDL‐cholesterol (mg/dl)	100.6 ± 29.7	96.6 ± 41.5	94.1 ± 52.8	77.6 ± 42.3	−15.52 (−39.11, 8.07)	.19
HDL‐cholesterol (mg/dl)	49.3 ± 11.8	47.0 ± 7.0	42.4 ± 6.8	42.4 ± 6.3	−2.57 (−6.65, 1.50)	.21
hs‐CRP (mg/L)	5.7 ± 2.2	5.7 ± 1.6	5.8 ± 2.6	4.9 ± 1.9	−0.89 (−1.50, −0.28)	.005
NO (μmol/L)	56.0 ± 6.1	56.6 ± 5.6	59.7 ± 5.1	58.6 ± 4.6	−0.51 (−3.13, 2.11)	.69
TAC (mmol/L)	1085.8 ± 106.6	1074.2 ± 112.3	1078.6 ± 202.9	1149.3 ± 128.2	72.28 (11.36, 133.19)	.02
GSH (μmol/L)	839.9 ± 144.4	851.3 ± 104.4	889.3 ± 97.4	957.5 ± 103.8	81.84 (15.40, 148.28)	.01
MDA (μmol/L)	4.7 ± 1.8	4.8 ± 1.2	4.7 ± 2.9	4.7 ± 1.8	−0.16 (−0.89, 0.56)	.64

*Note*: Data are mean ± SDs.

Abbreviations: FPG, fasting plasma glucose; GSH, total glutathione; HOMA‐IR, homeostasis model of assessment‐insulin resistance; HDL‐cholesterol, high density lipoprotein‐cholesterol; Hs‐CRP, high sensitivity C‐reactive protein; LDL‐cholesterol, low density lipoprotein‐cholesterol; NO, nitric oxide; QUICKI, quantitative insulin sensitivity check index; VLDL‐cholesterol, very low density lipoprotein‐cholesterol; TAC, total antioxidant capacity; MDA, malondialdehyde.

^a^ “Outcome measures” refers to the change in values of measures of interest between baseline and week 12. β (difference in the mean outcomes measures between treatment groups [NAC group = 1 and placebo group = 0]).

^b^ Obtained from multiple regression models.

## 4 DISCUSSION

Pharmacologic treatment options for many SUD are scarce. This is especially true for cannabis use and cocaine use disorder, for which there are not FDA‐approved (Tomko et al., 2018). NAC might reverse the neural dysfunction shown in SUD, as an over‐the‐counter antioxidant that affects the glutamatergic function in the brain; however, in current years, researchers have begun to tap its potential for treating psychiatric disorders and substance use (Roberts‐Wolfe & Kalivas, 2015). NAC is a generic medication with no known dependence liability, and a well‐established safety profile (known adverse reactions are generally mild). Notably, clinical side effects are not reported with the oral taking of NAC at doses up to 8000 mg/day (De Rosa et al., 2000). In this trial, we evaluated the effects of NAC consumption on psychological status and metabolic profiles in outpatients with SUD under MMT. We found that taking NAC for 12 weeks by people under MMT improved depression and anxiety symptoms, HOMA‐IR, and levels of hs‐CRP, GSH, and TAC concentrations; however, it did not have any effects on sleep quality, and other biochemical parameters.

### 4.1 Effects of NAC on psychological status

MMT programs may be correlated with psychological impairments such as poor general health, depression, and anxiety score (Peles et al., 2006; Yin et al., 2015). Depression and anxiety are classically considered one of the many components of the withdrawal syndrome in SUD (Baldwin et al., 2007; McClure et al., 2014). We demonstrated that taking NAC by the outpatients with SUD under MMT for 12 weeks improved depression and anxiety, but had no effect on sleep quality. Oral NAC is well tolerated and safe without any considerable side effects (Ooi et al., 2018). Current studies support its use as adjunctive therapy for psychiatric disorders and major mental disorders, administered concomitantly with existing medications, with a recommended dosage (of 2000 and 2400 mg/day) (Ooi et al., 2018; Zheng et al., 2018). In the last years, many evidence studies have indicated the usefulness of NAC consumption in treating clinical symptoms of substance abuse and dependence (Chang et al., 2021). In the study conducted by Cullen et al. (2018), NAC intake at a dosage of 600 mg twice daily (BID) (weeks 1−2), 1200 mg BID (weeks 3−4), and 1800 mg BID (weeks 5−8) improved global psychopathology scores and depression scores in young adults and female adolescents with nonsuicidal self‐injury. Also, one case study reported significant improvement as evidenced by a drop in clinical global impression severity (from 5 to 2) after 8‐week treatment with NAC (600 mg and increased to 1200 mg twice daily) in a 17‐year‐old adolescent boy with generalized anxiety disorder and social phobia (Strawn & Saldaña, 2012). It was recently reported that 1200 mg NAC daily in two divided doses for 12 weeks in methamphetamine‐addicted patients under methadone therapy had good efficacy on depression inventory, addiction severity, and craving index (Karami et al., 2020). However, in the meta‐analysis study, the results showed that prescribing NAC in mood disorders had no significant effect on depressive symptoms and manic signs as assessed by the Young Mania Rating Scale in bipolar disorder and only a small effect on depressive symptoms (Zheng et al., 2018). On the other hand, adjunctive NAC (3 g/day) for 20 weeks did not have any therapeutic effect on acute bipolar depression (Ellegaard et al., 2019). Presumed mechanisms of action of NAC may make clinical sense for its use in psychiatric disorders. Evidence from preclinical research indicates that NAC may modulate pathophysiological processes that are involved in multiple psychological, psychiatric, and neurological disturbances, including neurogenesis and apoptosis, oxidative stress, neuroinflammation, mitochondrial dysfunction, and dysregulation of dopamine and glutamate neurotransmitter systems (Dean et al., 2011; Deepmala et al., 2015). NAC is the precursor of the most abundant intracellular antioxidant, GSH, and acts as a direct free radical scavenger, hence playing a very active role in decreasing the oxidative injury. Also, NAC has been indicated to improve mitochondrial functioning in animal models of inflammatory bowel disease through the regeneration of mitochondrial membrane potential and hence decreases membrane permeability and apoptosis (Amrouche‐Mekkioui & Djerdjouri, 2012). In addition, NAC directly inhibits the inflammatory cytokines TNF‐*α*, IL‐1*β*, and IL‐6, at the protein and mRNA levels, in lipopolysaccharide‐activated macrophage cell lines along with a direct effect on brain macrophages through increased antioxidant properties, GSH production, and cysteine/glutamate exchange, hence regulating glutamatergic excitatory neuronal damage and redox reactions (Kigerl et al., 2012; Palacio et al., 2011). Moreover, NAC influences glutamatergic neurotransmission regulation of dopamine release from presynaptic terminals. NAC as a GSH precursor also acts as an important intracellular antioxidant, and hence seems to have a significant role in regulating dopamine‐induced neurotoxicity in different psychiatric disorders (Baker et al., 2002a, 2002b; Schulz et al., 2000). Furthermore, NAC is believed to have a role in metaplasticity and long‐term neuroadaptation that is potentially important in psychiatric disturbances (Moussawi et al., 2009; Reichel et al., 2011).

### 4.2 Effects of NAC on metabolic profiles

Our study indicated that taking NAC for 12 weeks in outpatients with SUD under MMT resulted in a significant reduction in HOMA‐IR, hs‐CRP levels, and a significant increase in GSH and TAC levels; however, it did not influence other metabolic profiles. Methadone therapy is linked to some disorders, including enhanced inflammatory cytokines levels (Chan et al., 2015). Also, there are reports that frequent use of methadone therapy and SUD are associated with DNA damage, lipid peroxidation, enhanced production of reactive oxygen species, and immune system disorders (Molavi et al., 2020; Vallecillo et al., 2018). Hypertension, impaired metabolism of insulin, overweight, and changes in lipids profiles in patients with SUD under MMT can result in the risk of drug‐related mortality, metabolic abnormalities, infectious complications, pulmonary diseases, and diabetes (Cousins et al., 2011; Maruyama et al., 2013; Vallecillo et al., 2018). Therefore, NAC therapies due to their useful impacts on the reduction of oxidative and inflammatory damage, and glycemic and lipid parameters might be useful for metabolic disturbances. Based on a meta‐analysis and a systematic review of clinical trials in Iran, TNF‐*α*, IL‐6, IL‐8, homocysteine, and MDA concentrations were improved in various diseases by NAC supplementation (Faghfouri et al., 2020). In addition, patients with community‐acquired pneumonia showed an improvement in MDA and TNF‐*α*, and TAC following 600 mg twice daily oral administration of NAC for 10 days (Zhang et al., 2018). In another study in women with polycystic ovary syndrome, consumption of NAC at a daily dosage of 1800 mg for 6 months substantially reduced BMI, FBS, fasting insulin, HOMA index, and LDL levels but did not affect triglyceride and total cholesterol (Javanmanesh et al., 2016). Also, in a meta‐analysis of 24 studies in 2020, Askari et al. examined the effects of NAC on serum levels of inflammatory biomarkers in adults. Results showed beneficial effects on CRP and IL‐6, but no effect on other inflammatory biomarkers (Askari et al., 2020). However, in a recent trial by Vural et al. (2018), daily prescription of NAC did not affect pro‐inflammatory mediators including IL‐6 and TNF‐*α* levels during 12 months in end‐stage renal disease patients. The particular characteristics of participants, different duration of the clinical trials, various dosages of NAC used, as well as differences in the research design could cause controversial data among the various reports. Several mechanisms have been reported showing the anti‐inflammatory effect of NAC: (1) NAC can inhibit NF‐κB, which plays a central role in the expression of genes involved in inflammation and oxidative stress; (2) NAC stimulates the synthesis of glutathione, which acts as a nucleophilic scavenger and as an anti‐oxidant in the event of oxidative tissue injury; (3) NAC might change the balance of T helper‐1 to T helper‐2 cells through its ability in changing the thiol intracellular solubility (Csontos et al., 2012; Kar Mahapatra et al., 2011; Sadowska et al., 2007). Also, NAC may ameliorate glycemic homeostasis and lipoprotein metabolism via regulating mRNA expression of peroxisome proliferator‐activated receptors and improving PI3K/Akt signaling pathway (Dludla et al., 2019).

The main strengths of the recent study were the use of a randomized double‐blind placebo‐controlled design and the fact that the planned study sample outpatients with SUD under MMT was achieved. In addition, not any changes in the type and dosages of MMT throughout the study period enhanced the generalizability and clinical utility of the results. However, several shortcomings of the clinical trials should be considered. First of all, the small size of investigated patients might be insufficient to show an effect of NAC administration in improved metabolic profiles and psychological scores in outpatients with SUD under MMT. Second, this trial had a relatively short duration of intervention. Third, we tested only one dose of NAC. Therefore, different doses of NAC should be examined, larger studies should be conducted with larger sample sizes and longer duration of intervention, as well as intervention in different addictions (e.g., cocaine, methamphetamine, and cannabis) are required to more precisely shed light. Also, we did not evaluate the cognitive function, relapses, craving, pain, and gene expression involved in metabolic profiles especially markers related to NF‐ĸB signaling pathways in outpatients with SUD under MMT. Unfortunately, because of limited funding, we did not examine the effects of NAC supplementation on these measures and it is suggested for a future study.

## 5 CONCLUSION

Overall, NAC consumption for 12 weeks had promising effects on mental health symptoms (depression and anxiety), insulin resistance, body anti‐oxidative defense system (TAC and GSH), and hs‐CRP levels in outpatients with SUD under MMT.

## AUTHOR CONTRIBUTIONS

Fateme Padoei, Peyman Mamsharifi, Pooya Hazegh, Homa Boroumand, Fatemeh Ostadmohammady, Samira Abbaszadeh‐Mashkani, Hamid Reza Banafshe, Amir Hassan Matini, Somayeh Ghadami Dehkohneh, and Amir Ghaderi contributed to the conception, design, statistical analysis, and drafting of the manuscript. All authors approved the final version for submission. Amir Ghaderi and Somayeh Ghadami Dehkohneh supervised the study.

## CONFLICT OF INTEREST

The authors declare that they have no conflict of interest.

### PEER REVIEW

The peer review history for this article is available at https://publons.com/publon/10.1002/brb3.2823.

## Data Availability

The datasets generated and/or analyzed during the current study are not publicly available because the intellectual property is owned by the funding body. They may be available from the corresponding author on reasonable request containing the approval from the associated funding body.
